# Stress, Burnout, Anxiety and Depression among Teachers: A Scoping Review

**DOI:** 10.3390/ijerph191710706

**Published:** 2022-08-27

**Authors:** Belinda Agyapong, Gloria Obuobi-Donkor, Lisa Burback, Yifeng Wei

**Affiliations:** Department of Psychiatry, University of Alberta, Edmonton, AB T6G 2B7, Canada

**Keywords:** teachers, stress, burnout, anxiety, depression

## Abstract

Background: Worldwide, stress and burnout continue to be a problem among teachers, leading to anxiety and depression. Burnout may adversely affect teachers’ health and is a risk factor for poor physical and mental well-being. Determining the prevalence and correlates of stress, burnout, anxiety, and depression among teachers is essential for addressing this public health concern. Objective: To determine the extent of the current literature on the prevalence and correlates of stress, burnout, anxiety, and depression among teachers. Method: This scoping review was performed using the PRISMA-ScR (Preferred Reporting Items for Systematic Reviews and Meta-Analyses extension for Scoping Reviews). Relevant search terms were used to determine the prevalence and correlates of teachers’ stress, burnout, anxiety, and depression. Articles were identified using MEDLINE (Medical Literature Analysis and Retrieval System Online), EMBASE (Excerpta Medica Data Base), APA PsycINFO, CINAHL Plus (Cumulative Index of Nursing and Allied Health Literature), Scopus Elsevier and ERIC (Education Resources Information Center). The articles were extracted, reviewed, collated, and thematically analyzed, and the results were summarized and reported. Results: When only clinically meaningful (moderate to severe) psychological conditions among teachers were considered, the prevalence of burnout ranged from 25.12% to 74%, stress ranged from 8.3% to 87.1%, anxiety ranged from 38% to 41.2% and depression ranged from 4% to 77%. The correlates of stress, burnout, anxiety, and depression identified in this review include socio-demographic factors such as sex, age, marital status, and school (organizational) and work-related factors including the years of teaching, class size, job satisfaction, and the subject taught. Conclusion: Teaching is challenging and yet one of the most rewarding professions, but several factors correlate with stress, burnout, anxiety, and depression among teachers. Highlighting these factors is the first step in recognizing the magnitude of the issues encountered by those in the teaching profession. Implementation of a school-based awareness and intervention program is crucial to resolve the early signs of teacher stress and burnout to avoid future deterioration.

## 1. Introduction

The teaching profession can be highly stressful, and this stress may lead to reduced job satisfaction, burnout, and poor work performance. Stress is a normal response to upsetting or threatening events and becomes pathological when chronic [1]. Chronic stress can impede day-to-day functioning and emotional balance, and it is a risk factor for developing other psychiatric illnesses, such as anxiety and depression [1,2,3]. Prolonged teacher stress negatively correlates with job satisfaction and positively correlates with intending to leave the teaching profession. It may also result in withdrawal behaviour, including physically or psychologically leaving the work setting [4,5]. Chronic stress may also lead to inappropriate anger and increased alcohol and drug consumption [6,7], and it can cause an individual to experience excessive anxiety, mental fatigue, and burnout, while also predicting increased depression [8,9,10]. According to Maslach, stress occurs when a person perceives an external demand as exceeding their capability to deal with it [11]. Teacher stress can be associated with demoralization, and a disrupted sense of self-consistency [8,9]. Canadian teachers, like their global counterparts, also experience high-stress levels. A study by Biron et al. showed that the proportion of Quebec teachers who reported a high level of psychological distress was twice as high (40%) as that reported for a Quebec-wide general population sample (20%) [12]. During the COVID-19 pandemic, survey results indicated that nearly 70% of respondents worried about their mental health and well-being [13]. Meanwhile, a cross-sectional study showed that two-thirds of teachers perceived stress at work at least 50% of the time [14]. Teacher workload is one of the most common sources of stress [15]; however, there is a lack of systematic understanding about how stress is measured, its prevalence globally, what factors lead to stress and what causes the associated negative outcomes among teachers.

Burnout is considered a stress-related problem for individuals who work in interpersonally oriented occupations such as healthcare and education [16,17]. According to Shukla et al., burnout among professionals such as teachers can result from excessive demands on their energy, strength and resources [7]. There is increasing evidence that burnout as a negative stress response represents a risk factor not only for depression but also for cardiovascular and other somatic diseases [17]. Researchers conceptualize burnout as having three interrelated components: emotional exhaustion, depersonalization, and reduced personal accomplishment [6,7,11,16]. Emotional exhaustion represents emotional depletion and a loss of energy. Depersonalization is the interpersonal dimension of burnout. It refers to a negative, callous, or excessively detached response to other people. There is evidence that job satisfaction is negatively associated with emotional exhaustion and positively associated with self-perceived accomplishment, but not significantly related to cynicism [18]. Additionally, reduced accomplishment describes the self-evaluation dimension of burnout, including feelings of incompetence and a lack of achievement and productivity at work [6,16,18,19]. Mild burnout involves short-lived irritability, fatigue, worry, or frustration. Moderate burnout has the same symptoms but lasts for at least two weeks, whereas severe burnout may also entail physical ailments such as ulcers, chronic back pain, and migraine headaches [20]. Research suggests that workplace improvements to reduce burnout could prevent adverse sequelae, improve health outcomes, and reduce healthcare expenditures [21]. More systematic research is needed to further understand the factors in the workplace to address burnout and improve teacher health outcomes.

Anxiety and perceived stress are predicted by workload, student behaviour, and employment conditions [22]. According to Kamal et al., a considerable lack of administrative support is the single biggest factor increasing anxiety [23]. Those with low job satisfaction are more susceptible to experiencing burnout, high anxiety levels and depression [24,25]. Teacher stress contributes to teacher anxiety and may trigger anger, further intensifying anxiety [5,26]. The published literature shows that participants who reported high anxiety levels also reported high burnout levels [27]. Moreover, some studies report a very high prevalence of stress (100%), anxiety (67.5%), and depression (23.2%) among teachers [28], prompting calls for research and interventions to address this critical issue [23]. Despite this, more research is needed to understand what factors play key roles in triggering anxiety symptoms among educators and how stress, burnout, anxiety, and depression relate to each other.

Depression can lead to numerous deficiencies and is considered the worldwide primary cause of work disability [29,30]. Depression among teachers can also significantly impact their health, productivity, and function [31], with particularly pervasive effects on personal and professional life [32]. Individuals with depression often experience difficulties meeting interpersonal, time-management, and productivity demands. They may also encounter psychological problems, decreased work quality, absences due to illness, and increased work disability, all of which can profoundly impact worker productivity [30,31,33]. One study found that teachers’ most robust major depressive disorder (MDD) predictors included a low job satisfaction, high perceived stress, somatization disorder, and anxiety disorder [31]. Like with anxiety symptoms, more research is needed to understand what factors play key roles in triggering depression symptoms among educators and how depression relates to other psychological conditions including stress, burnout, and anxiety.

Currently, the authors are planning a study to assess the prevalence and correlates of stress, burnout, anxiety, and depression among elementary, junior high and high school teachers in Alberta and Nova Scotia, Canada [34]. This planned study will also evaluate the effectiveness of a daily supportive text message intervention, the Wellness4Teachers program, to address stress, burnout, anxiety, and depression among elementary and high school teachers in Canada [34]. Within this context, this scoping review aims to identify and summarize the literature on the prevalence and correlates of teachers’ stress, burnout, anxiety, and depression and to determine the problem’s extent in different jurisdictional contexts. The review also aims to identify the gaps in knowledge for future research. Identifying the correlates of these emotional and mental conditions may also facilitate the research and development of early interventions which can be implemented to address this phenomenon.

## 2. Methods

### 2.1. Study Design

This scoping review was planned and conducted in adherence to the Preferred Reporting Items for Systematic Reviews and Meta-Analyses extension for Scoping Reviews (PRISMA-ScR) statement [35]. We adopted a comprehensive search strategy that allows replicability, reliability, and transparency. This scoping review also followed Arksey and O’Malley’s five-stage approach to scoping reviews: identifying the research question, searching for relevant studies, the study selection, charting the data, and collating, summarizing and reporting the results [36].

### 2.2. Developing the Research Question

Our research question was: “What are the prevalence and correlates of primary and secondary teachers’ stress, burnout anxiety and depression in different jurisdictions?”

### 2.3. Information Sources and Search Strategy

The search was performed by using relevant terms to identify and select articles in the following databases: MEDLINE (Medical Literature Analysis and Retrieval System Online; Ovid MEDLINE ALL), EMBASE (Excerpta Medica Database; Ovid interface), APA PsycINFO (Ovid interface), CINAHL (Cumulative Index of Nursing and Allied Health Literature) Plus with Full Text (EBSCOhost interface), Scopus Elsevier and ERIC (Education Resources Information Center (EBSCOhost interface). The search consisted of keywords representing the concepts of stress, burnout, depression and anxiety among teachers and their correlates and prevalence. The specific MeSH terms, keyword and descriptors included: (depress* OR depression OR “depressive disorder” OR “depressive symptoms” OR “major depressive disorder” OR anxiety OR “anxiety disorder” OR “generalized anxiety disorder”) AND (burnout OR “burn out” OR stress OR “occupational stress” OR “mental exhaustion” OR “emotional exhaustion”) AND (teacher* OR educator* OR tutor* OR schoolteacher* OR “school teacher*”). The database search was completed on the 20th of February 2022.

### 2.4. Selection of Studies

**Figure 1:**
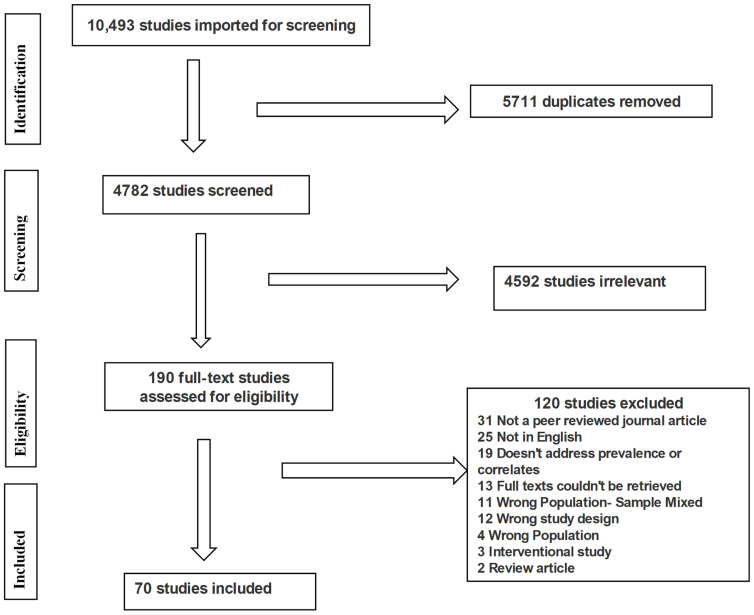
PRISMA flow chart.

The search strategy was developed based on specific inclusion criteria. Articles were considered eligible for inclusion in this scoping review if they addressed either the prevalence or correlates of burnout, stress, depression, or anxiety among teachers or educators. The articles were limited to original, peer-reviewed quantitative articles written in English. Articles were excluded from the review if the study participants were tertiary or university teachers or students. Studies on interventions’ outcomes, case reports, meta-analyses, systematic reviews, opinion pieces, commentaries, editorials, or grey literature such as non-peer-reviewed graduate student theses, non-research articles or conference reports were excluded. The search was not limited by publication year. Two researchers independently reviewed the citations during the title, abstract screening, and full-text review phase. All discrepancies were resolved through discussion and consensus. We identified 190 articles for full-text review, of which 120 articles were excluded. The PRISMA flow diagram summarizes this information in detail (Figure 1).

### 2.5. Data Charting and Extraction Process

The research team extracted data for each selected article according to the following domains: author(s) name, year of publication, country of study, study design, assessment tools used, sample size (N), age, main findings, and conclusion.

### 2.6. Collating, Summarizing, and Reporting the Results

This study presents an overview of existing evidence relating to the prevalence and the correlates of stress, burnout, anxiety, and depression among teachers. All the relevant data were organized into tables and validated by at least two team members. The characteristics and results reported in each included article were summarized. In addition, the prevalence range for the psychological conditions in high-quality studies were determined after identifying the high-quality studies for each psychological condition in this scoping review using the Joanna Briggs Institute’s (JBI) critical appraisal checklist for prevalence studies [37]. The JBI checklist includes: studies with an adequate sample size, studies which provided an appropriate sample frame to address the target population, studies with an adequate response rate, studies which had a high response rate, studies in which a systematic approach was used for the data capture to ensure the study sample was representative of the study population, and studies with an adequate statistical analysis.

## 3. Results

### 3.1. Study Characteristics

**Figure 2:**
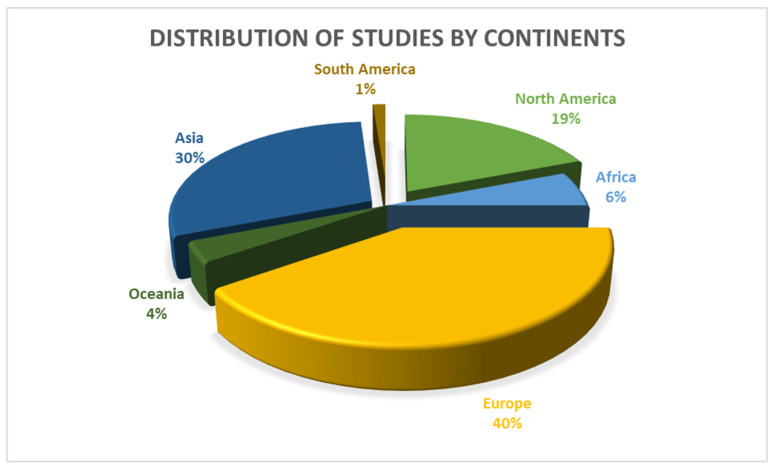
Summary of studies by continents.

The search strategy identified 10,493 citations. Covidence software [38] was used to automatically remove 5711 duplicates. One hundred and ninety articles remained for a full-text screening, and seventy of these were eligible for inclusion. Overall, 67 articles were quantitative cross-sectional studies. One study was a mixed quantitative and qualitative study, and two studies were randomized controlled trials. The seventy articles included a total of 143,288 participants, who were all teachers. The sample size for an individual article ranged from 50 to 51,782 participants, with an age range from 18 years to 75 years. The minimum response rate was 13% and the maximum was 97.4% with the median response rate of 77%. The articles included studies from 1974 to 2022. Most studies (79%) were published between 2007 and 2022, and 21% were from 1974 to 2006. Most of the studies were conducted in Europe (40%), followed by Asia (30%) and North America (19%). In contrast, African, South America and Oceanian studies represented 6%, 1% and 4%, respectively, as shown in Figure 2. One study [39] was conducted across multiple continents.

**Figure 3:**
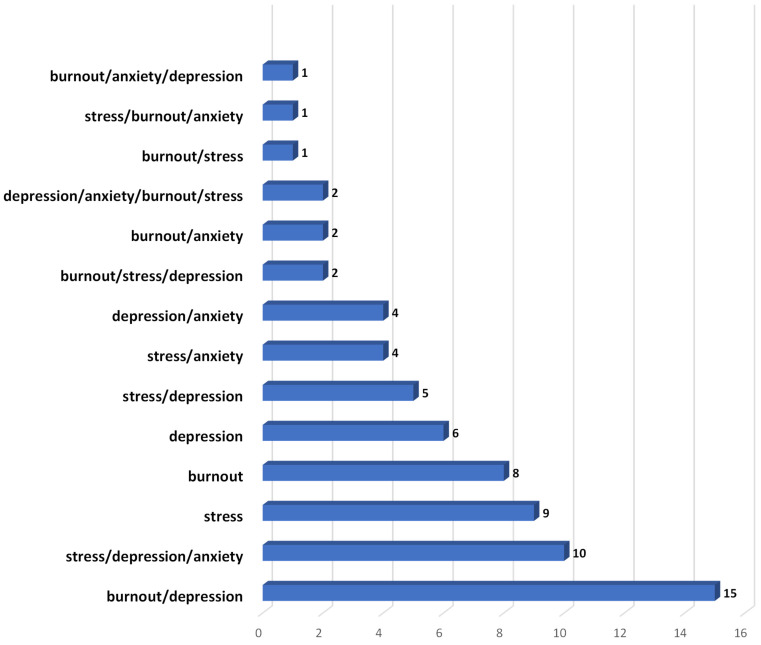
Distribution of stress, burnout, anxiety and depression among the included studies.

From Figure 3: Most studies reported on multiple outcomes, indicating the interrelatedness of stress, burnout, anxiety, and depression. Some articles reported on a single outcome, such as stress (N = 9), burnout (N = 8), or depression (N = 6). Burnout and depression (N = 15), stress and depression (N = 5), burnout and anxiety (N = 2), anxiety and depression (N = 4), and stress and anxiety (N = 4), were commonly paired outcomes. One study (N = 1) specifically examined the paired outcomes of burnout and stress. In addition, the outcome of the interaction between three or four of these psychological problems were explored by some studies: anxiety, depression, and stress (N = 10); anxiety, burnout and depression (N = 1); stress, burnout and anxiety (N = 1); stress, burnout, and depression (N = 2). Finally, two articles reported the interaction between stress, burnout, anxiety, and depression.

**Figure 4:**
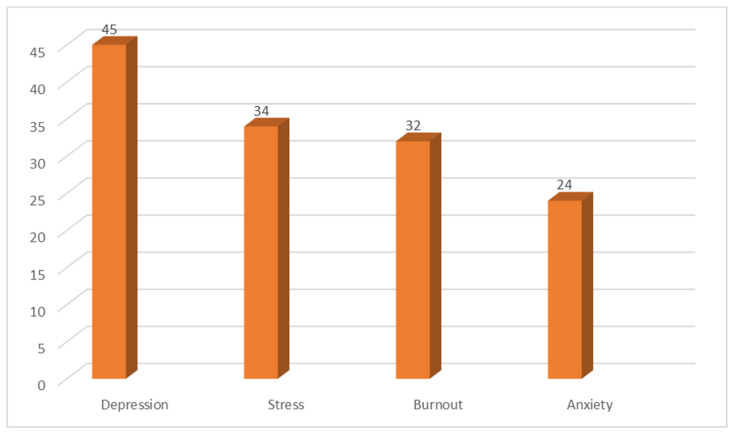
Number of studies reporting each psychological problem.

Figure 4 shows that depression was the most reported psychological problem among the included studies and the least reported was anxiety.

Most of the articles (27 of 32; 84%) used Maslach’s Burnout Inventory to explore the three interrelated components of burnout. Five of thirty-two (16%) studies used the Oldenburg Burnout Inventory, the Shirom–Melamed Burnout Inventory, or the Teacher Burnout Scale. The most frequently utilized scales for measuring depressive or anxiety symptoms (55 studies) were the Center for Epidemiological Studies Depression Scale (CES-D) (N = 14; 25%), Depression, Anxiety and Stress Scale (DASS), (N = 10 18%), the Patient Health Questionnaire-9 (PHQ-9), (N = 9; 16%), and the Beck Depression Inventory (BDI), (N = 6; 11%). The less popular scales included the Goldberg Anxiety and Depression Questionnaire, COVID-19 Anxiety Scale, Zung Self-Rating Depression Scale (SDS), and the Manifest Anxiety Scale. For the 29 studies measuring stress, the most common scales utilized were the (DASS) (N = 9; 31%), the Teacher Stress Inventory (N = 5; 17%), and the Perceived Stress Scale (PSS) (N = 3; 10%). Other scales included: the Occupational Stress Inventory, Job Stress Inventory, Ongoing Stressor Scale (OSS), Episodic Stressor Scale, and Bruno’s Teacher Stress.

### 3.2. Prevalence and Correlates of Burnout, Stress, Anxiety and Depression

The prevalence and correlates of stress, burnout, anxiety, and depression as identified in the literature search are summarized in Table A1 and Table A2 in Appendix A.

### 3.3. Prevalence of Stress

The reported stress prevalence rates were heterogenous, which may reflect, in part, the use of different stress measures. The prevalence of stress in all forms ranged from 6.0% to 100% [28,40], with a median of about 32.5%. In addition, the lowest, highest and median stress prevalence ranges from 2020 to 2022 (after the pandemic and lockdown) were, respectively, 6.0% [40], 66.0% [41] and 10.7%. Similarly, the lowest, highest and median stress prevalence up until 2019 (prior to the pandemic and lockdown) were, respectively, 7.0% [42], 100% [28] and 33.9%.

Early studies of teacher stress found a relatively high degree of stress. For example, 76% [43] and 87.1% [44] of teachers described their stress levels at their school as moderate or significant, respectively. In some studies, 45.6% reported “much stress” [44] or “almost unbearable” stress (20%) [43]. Another study echoed these findings, reporting 32% ‘slightly’ stressed and 67% ‘extremely’ stressed teachers, with only 1% indicating no stress [45].

Earlier studies on teacher stress are consistent with more recent findings, indicating teacher stress is a long-standing issue and is challenging to tackle. A 2021 study completed during the COVID-19 pandemic reported a 6.0% prevalence of severe to highly severe stress among teachers [40]. This is similar to another recent but pre-pandemic study which reported a 7.0% prevalence of “severe to extremely severe” stress, a 32.3% prevalence of stress, and 25.3% prevalence of mild to moderate stress [42].

### 3.4. Prevalence of Burnout

Published studies have identified three different burnout profiles among teachers with the prevalence ranging from 25.12% to 48.37% [11,46]. These are, (1) groups of teachers with predominantly low levels of emotional exhaustion and high levels of personal accomplishment, (2) teachers with high levels of emotional exhaustion and depersonalization, and (3) teachers with low levels of depersonalization and personal accomplishment [46]. These groups show the combination of the three interrelated components of burnout reported by Maslach et al. [6,7,11,16].

Variable prevalence of burnout and psychological distress have been reported among teachers [47], with the burnout prevalence at all levels ranging from a low of 2.81% [7] to a high of 70.9% [48], with a median of 28.8% (Table A1). The lowest, highest and median burnout prevalences from 2020 to 2022 (after the pandemic and lockdown) were, respectively, 3.1% [48], 70.9% [48] and 27.6%. Similarly, the lowest, highest and median burnout prevalences up until 2019 (prior to the pandemic and lockdown) were, respectively, 2.81%, 63.43% [7] and 25.09%.

In an early study, only 11% of the teachers were classified as burnt out, and more than half (68.5%) of the teachers reported they did not experience any burnout [49]. Some studies reported burnout prevalence in the three subdimensions [50]. For instance, four studies reported a burnout prevalence of 11% to 40% for emotional exhaustion, depersonalization and for reduced personal accomplishment [3,46,49,50]. Studies have also reported that 18.3% to 34.9% of teachers may be at risk of or are threatened by burnout syndrome [3,25,51]. Higher burnout scores and subdimensions such as emotional exhaustion and depersonalization burnout were significantly higher among female teachers than male teachers [51,52,53]. Likewise, a higher percentage of males (59.38%) showed low burnout than did females (53%) [54]; however, other studies have reported contradictory results where males had a slightly higher burnout prevalence of 56.0% than females of 53.0% [55] and 31.88% of males and fewer females (25%) reported a lack of personal accomplishment [54].

There are also studies reporting various levels of burnout ranging from low/no burnout (58.12%) to moderate (2.81% to 70.9%) and severe levels of burnout (3.1% to 33.3%) [7,25,47]. Regarding the subjects taught by teachers, science stream and science teachers reported experiencing slightly more burnout (14.38% to 26.26%) than arts stream and art teachers, who reported an average burnout prevalence of 12.5% to 25% [7].

### 3.5. Prevalence of Anxiety

The anxiety symptoms prevalence ranged from 4.9% to 68.0% [42,56], with a median prevalence of 26.0%. Furthermore, the lowest, highest, and median anxiety prevalences from 2020 to 2022 (after the pandemic and lockdown) were, respectively, 10.5% [57] 66.0% [41] and 38.9%. Similarly, the lowest, highest, and median anxiety prevalences up until 2019 (prior to the pandemic and lockdown) were, respectively, 7.0% [28], 68.0% [42] and 26.0%.

Early studies indicated that teachers’ anxiety prevalence ranged from 26% for borderline anxiety, 36% for minimal or no anxiety, and 38% for clinically significant anxiety [45]. Recent studies have reported a similar prevalence for low anxiety at 17.6%, mild at 23.2% [28] and 7.0% to 23.3% for severe to extremely severe anxiety [28,39,41]. Another study reported an anxiety prevalence of 43% among teachers. The prevalence of anxiety did not change significantly during the COVID-19 pandemic, with most teachers (56.2%) reporting no change in their anxiety during the pandemic compared with before the pandemic, and only 4.9% of teachers reported an increase in anxiety levels from the baseline during the first week of the 2020–2021 school year [58].

### 3.6. Prevalence of Depression

The prevalence of depression among teachers ranged from 0.6% to 85.7% [48,59], with a median of 30.7%. The lowest, highest, and median depression prevalences from 2020 to 2022 (after the pandemic and lockdown) were, respectively, 0.6% [48], 85.7% [59] and 23.5%. Similarly, the lowest, highest and median depression prevalences up until 2019 (prior to the pandemic and lockdown) were, respectively, 0.7% [28], 85% [60] and 24.1%.

Early studies showed a highly varied prevalence of depression, with 79% of teachers scoring at the low or no depression levels in one study. This study also reported that 17% of teachers had borderline depression scores, and 4% had scores that indicated clinical depression [45]. Studies from 2008 onwards identified that the prevalence of depression ranged from 17.86% to 49.1% [3,41,55,60,61] and the prevalence of severe to extremely severe depression ranged from 0.7 to 9.9% [42], whilst the prevalence of mild depression ranged from 20 to 43.9% [41,42,60,62]. Soria-Saucedo et al. reported a particularly high prevalence (16%) of severe depression symptoms among teachers [61]. Depression was also found to range from 45% to 84.6%, depending on the educational level and teaching experience, and was highest among those with a lower education level, followed by teachers with more teaching experience [42].

Studies during the pandemic demonstrated higher rates of mild depression but similar rates of severe depression symptoms among teachers. In one study, 58.9% of teachers had mild depression, 3.5% had moderate, and 0.6% had severe depression. [48]. Another study reported that 3.2% of teachers had severe to extremely severe depression [40]. According to Keyes, ‘flourishing’ denotes being filled with positive emotion and functioning well psychologically and socially while ‘languishing’ in life signifies the individual has poor mental health with low well-being [59,62]. Capone and Petrillo reported that 38.7% of ‘flourishing’ teachers reported a lower prevalence of depression but higher levels of job satisfaction. A severe rating of depression was also reported by 85.7% of ‘languishing’ teachers [59].

### 3.7. Prevalence Range and Median for Stress, Burnout, Anxiety and Depression Reported in High Quality Studies

After applying the JBI checklist [37] to identify high-quality studies, the clinically meaningful (moderate to severe) burnout among teachers recorded by three studies ranged from 25.12% to 74% [25,46,47]. Similarly, three studies reported stress at clinically meaningful levels which included severe, extremely severe, moderate to high or very stressful, and a great deal of stress, with a prevalence ranging from 8.3% to 87.1% [43,44,57]. Likewise, two studies reported the prevalence of clinically meaningful anxiety among teachers ranging from 38% to 41.2% [45,57]. Furthermore, five studies [44,47,57,63,64] reported the prevalence of depression in clinically significant levels, which included terminologies such as major, moderate, moderate to severe, and extremely severe depression symptoms. The lowest prevalence in this category was 4% [45] and the highest category was 77% [65]. Finally, the median prevalence of stress, burnout, anxiety, and depression among these studies were, respectively, 67.0%, 60.9%, 39.6%, and 14.%.

### 3.8. Correlates of Stress, Burnout, Anxiety and Depression

The correlates of stress, burnout, anxiety, and depression, as extracted from Table A1 and Table A2, are summarized in Table 1. A wide range of variables are significantly associated with teachers’ stress, burnout, anxiety and depression and can be divided into socio-demographics, school, organizational and professional factors, and social and other factors, including intrapersonal factors. The most reported correlates were sex, age, gender, marital status, job satisfaction, subject taught and years of teaching [28,40,57,63,66,67]. Socio-demographic factors, such as age and sex, and work-related factors correlate with depression, anxiety and stress [42]. Emotional exhaustion is correlated with age, gender and marital status. [39,52,53,68]. Other studies, however, refute these, indicating that no significant demographic variable correlations were found between burnout and depression, and that depressive symptoms in men and women were similar [64,69]. Capone et al. also noted that all the school climate factors, such as social support, were negatively related to depression [70]. Higher levels of co-worker support were related to lower levels of anxiety and depression [71].

Organizational factors associated with anxiety included: work overload, time pressures causing teachers to work during their free time, and role conflict. There were significant correlations between the reported anxiety and those stressors relating to pupils and parents [45]. In addition, interpersonal conflict, organizational constraints and workload were reported to result in depression through increasing job burnout [73]. Furthermore, depressive symptoms correlated with teaching special needs students and had a significant and robust relationship with the general burnout factor [50]. Self-perceived accomplishment was also positively associated with autonomy and negatively associated with low student motivation [18]. Personal accomplishment had a significant positive relationship with the number of teaching hours per week [40]. On the contrary, a cross-sectional study by Baka reported that increased work hours are usually accompanied by job demands, job burnout, and depression [73]. Job strain, job demand and job insecurity all showed positive associations with depressive symptoms [80,94]. Work-related factors, such as workload, were also correlated with stress, burnout, depression, and anxiety [42,73].

Furthermore, the educational level and teaching experience also predict depression. Depression was highest among teachers with a lower education followed by teachers with the most teaching experience [42]. Teacher stress was reported to be significantly associated with psychological distress, and social support could moderate the influence of stress; hence, the high-stress and the low-support group were most vulnerable to anxiety [74]. Studies have also reported that 55% of teachers without spousal support had depression [42]. In addition, stress was reported to be associated with missed work days, high anxiety and high role conflict [43,89] and 53.2% of teachers identified work as a source of long-term stress, leading to burnout [55]. According to Fei Liu et al. resilience significantly correlated with job burnout and turnover intention, and low resilience could result in a high job burnout [86]. The research also showed that personality trait neuroticism was the best predictor of burnout (28–34%) [67].

### 3.9. Association between Stress, Burnout, Anxiety and Depression

A significant overlap was reported between stress, burnout, anxiety and depression. Eighteen articles reported a correlation between burnout and depression, with differences in depressive symptomatology depending on the prevalence of burnout [3,18,25,41,42,48,50,52,54,60,64,69,84,86,92,95]. Three articles reported a correlation between burnout and anxiety symptoms [52,64]. Seven articles reported a correlation between stress and anxiety [28,58,65,71]. Six articles reported a correlation between stress and depression [28,31,43,61,68,71]. A correlation exists between moderate depressive disorder and anxiety disorder as well as stress [31,96]. Negative affectivity (a tendency to feel depression, anxiety, or stress) plays a role in the development of burnout among teachers. Teachers who developed a more markedly negative affectivity also felt more burnt out, and the opposite was true [41]. This may be related to rumination. According to Nolen-Hoeksema, rumination is a pain response which entails a recurrent and passive focus on the symptoms of pain and their likely causes and outcomes [97]. Ruminative responses may prolong depression by overly focusing on negative thinking and may affect one’s behaviour and problem-solving [97]. Liu et al. reported that rumination moderated the association between job burnout and depression and that burnout was a stronger predictor of depression in teachers who experienced low rumination rather than high rumination [98]. This was explained by the importance of rumination for depression; with an improvement in the rumination level, job burnout had less ability to predict depression for those with high rumination levels.

There is a strong association between burnout and depression, as reported in several studies. High frequencies of burnout symptoms were identified among clinically depressed teachers [92], with 86% to 90% of the teachers identified as burnt out meeting the diagnostic criteria for a depressive disorder [60,64], mainly for major depression (85%) [60]. In 25% to 85% of teachers with no burnout, depression ranged from 1% to 15% of the study sample. Specifically, only 1% to 3% of the participants in the no-burnout group were identified as having minor depression or depression not otherwise specified (2%) [60,64]. A history of depression was reported by about 63% of the teachers with burnout and 15% of the burnout-free teachers [60]. The high overlap between depression and burnout was emphasized in one study, which categorized depression as “low burnout-depression” (30%), “medium burnout-depression” (45%), and “high burnout-depression” (25%) [92]. Notably, the report suggests that although teacher burnout leads to subsequent depressive symptoms, it is not true vice versa [95]. Furthermore, burnout symptoms at ‘time one’ did not necessarily predict depressive symptoms at ‘time two’ [99]. Another study reported a positive relationship between burnout and depression [84]. This was confirmed by a study which suggested that depressive symptoms had a significant and robust association with the general burnout factor [50].

Anxiety disorder is also associated with higher perceived stress and major depression [65]. In one study, higher ongoing stressors were positively associated with higher anxiety levels. Continuous and episodic stressors were significantly and positively associated with anxiety and depression. They accounted for 28% (adjusted 25%) of the variability in anxiety and 27% (adjusted 24%) of the variability in depression. [71]. In contrast, higher levels of co-worker support were related to lower levels of anxiety and depression [71]. Teachers reported a high prevalence of depressive symptomatology relating to subjective and school-related stress [43].

## 4. Discussion

This scoping review included 70 articles. The prevalences of stress, burnout, anxiety and depression reported in this scoping review are similar to those reported in two systematic reviews and meta-analysis conducted among teachers during the pandemic. For example, the prevalence of stress reported by Ma et al., from a meta-analysis of 54 studies was 62.6%, whereas the prevalence of anxiety was 36.3% and depression was 59.9% among teachers during the pandemic [100]. In another meta-analysis, the prevalence range of anxiety was 10% to 49.4%; depression was 15.9% to 28.9%; and stress was 12.6% to 50.6% [101], which all fall within the range reported in this scoping review for stress [28,40], anxiety [42,56], and depression [48,59]. However, the minimum in all cases was higher during the pandemic, suggesting an increase in psychological problems during the pandemic.

The varying prevalence for stress, burnout, anxiety and depression reported by different studies in this review may be attributable to heterogeneous study designs, including the sample size, location, period of data collection, diversity in the standardized scales used for the assessment, and other factors such as the class size and grade taught [102,103]. In this scoping review, the studies used combinations of terminologies such as “none,” “slightly,” “significant,” “much,” “extremely,” “considerably”, “almost unbearable”, “quite a bit” or “a great deal” to describe the level of stress experienced by teachers according to the measures utilized,, such as the Teachers Stress Inventory [44,77] or the Bruno Teachers Inventory [43]. The prevalence rates also varied with population, for example, in the case of Fimian, the teachers were teaching special needs students, and this may explain the relatively high prevalence (87.1%) recorded [44]. More recent studies which used other scales, such as the Perceived Stress Scale (PSS), and the Depression Anxiety Stress Scales (DASS), used terminologies such as “symptoms of stress”, ranging from “mild,” “moderate,” “mild to moderate” or “extremely severe”, to describe the stress levels. For burnout, although most studies used a combination of the three interrelated components of burnout reported by Maslach et al. [6,7,11,16], some studies focused on reporting the sub-dimensions of burnout, whilst others reported general burnout. Varying expressions such as “low burnout”, “high burnout, “severe burnout”, and moderate were used to describe burnout, making it difficult to make an effective comparison. It was also not clear whether the stress and burnout experienced by the participants were everyday existential life experiences that everyone faces or chronic ones that needed intervention, as these were not specifically stated in the studies. It is essential that future research clarifies this to estimate their prevalence rates more accurately. Secondly, as indicated in the review, the studies applied various scales to measure the prevalence of psychological disorders; however, there was a lack of consensus. This scoping review provides a comprehensive picture of the prevalence of the target outcomes and sets up a foundation for future systematic reviews and meta-analysis to accurately estimate the prevalence of these outcomes among teachers.

The essential correlates of stress, burnout, anxiety, and depression identified in this review include socio-demographic factors such as sex, age, gender, marital status, school (organizational) factors and work-related factors (years of teaching, class size, job satisfaction, subject taught and absenteeism). Most studies were published in the last fifteen years (2007–2022), indicating a recent increase in interest in this area of research.

### 4.1. Socio-Demographic, School and Work-Related Factors as Determinants of Stress

Socio-demographic factors such as sex, age and marital status significantly influence teacher stress [54]. Sex correlates with stress although there are some conflicting reports [42,53,76], especially between the levels of stress experienced by males and females. Some studies suggest that female teachers experience more stress than their male counterparts [28,75,77]. Working women often have additional demands at home, and trying to accomplish both roles may increase their stress levels [104] compared to males who may have less demand from home. However, this may be context-dependent, as no sex difference in occupational stress was reported among police officers [105], for example. The demand from female teachers’ personal lives, including marital issues and home, may be a source of increased stress levels [104]. Among the general workforce, work–family conflict has been reported to be significantly associated with work stress [106], and this is not confined only to females. This argument is confirmed in three separate studies, which reported that gender, per se, was not a significant predictor of perceived stress [39,85,89]; thus, it is possible that these differences may, rather, be due to differences in the scales used or the effect of organizational factors. For example, the organisational effect experienced by female teachers in a female only elementary or high school may differ from that experienced in a male only or mixed sex teaching environment; however, further research is needed in this area of gender influencing stress factors. Findings from the Canadian Community Health Survey data nonetheless endorsed a difference between males and females regarding work stress, in particular supervisor support. Higher levels of supervisor support seemed to lower work stress amongst women but not men [107]. Among the general population, social support at work could be more strongly related to a stress reduction in women than in their male counterparts [108] Sex difference was also observed in relation to student behaviour, with women experiencing increased stress [42,77]. In particular, female teachers’ collective efficacy and beliefs about their school staff group capabilities may lower their stress from student behaviour. Findings from the study by Klassen support the hypothesis that teachers’ collective efficacy serves as a job resource that mediates the effect of stress from student behaviour [77]. Interventions addressing gender/sex differences may also be considered in supporting female educators’ mental health and work productivity.

A study among refugee teachers also endorsed sex differences in stress [42,57]; however this was in relation to self-care and the association was moderated by age [57]. Higher occupational stress scores were observed among teachers over 40 years [28]; nonetheless, among the general population, the published literature reports that the ageing process can worsen or counter the effects of stress [109], indicating that age does not necessarily increase stress. The cause of increased stress, hence, shifts to other factors such as the poor academic performance of students, or a lack of assistance [78], which may be influencing an increase in stress.

The class size, grade level taught, workload, poor student performance or lack of progress and other work and school-related factors contribute to teachers’ stress. According to Fimian et al., when stressful events or the perception of them are not ultimately resolved or improved, this may result in several physiological manifestations [44]. There is clear data indicating that teacher stress was intensified among primary school teachers, special needs teachers, and teachers in private schools who provided more support and input to students than other teachers [28,78,85,110]. The additional time and energy teachers may invest in primary school kids, who are usually much younger and may require more support, may explain the increased stress among primary school teachers. Again, teaching special needs students may require significant teacher input and assistance, depending on the nature and degree of the disabilities. There is also an increased expectation from teachers in private schools regarding the students’ performances, leading to increased stress [28]. A study conducted among primary and secondary school teachers in Pakistan concluded that government school teachers were more satisfied with their working conditions than private school teachers [110], and thus, may experience less stress. In addition, the school location (rural vs. urban), teacher role ambiguity and coherence further exacerbated teacher stress [3,75,89,111]. An excessive use of technological devices, such as mobile phones, has also been associated with social disruption [112] and may result in a lack of concentration or poor student performance at school [112,113], leading to teacher stress. Teachers experiencing more significant stress were also burnt out [68]. For example, during the pandemic, teachers had to adopt and adjust to teaching online, and virtual instruction teachers had the most increased anxiety [58]. Nonetheless, a rapid systematic review with a meta-analysis reported that teacher stress during the pandemic was still comparatively lower in school teachers with a prevalence of 13% ([95% CI: 7–22%]) in comparison to studies with university teachers as the participants of 35% ([95% CI: 12–66%]) [114].

While there are complex interactions among several factors which contribute to teacher stress, there have been limited evidence-based interventions to help teachers alleviate these stress sources despite some self-reported coping strategies. This research gap started to receive attention during the COVID-19 pandemic through the application of mindfulness-based interventions [115], warranting more advanced research on how to best address these challenges in education.

### 4.2. Socio-Demographic, Years of Teaching, School and Work-Related Factors as Determinants of Burnout

Burnout continues to pose problems within the teaching profession, and factors such as gender, sex, age, marital status and the number of years teaching correlated with the degree of burnout [40,47,51,52,53,54,55,63,67,68,72,73], although conflicting results were reported with potentially different explanations. Differences in the study design, particularly the scales used to assess burnout as well as geographical and organizational factors, may account for some of the conflicting results. In addition, there could be an interplay between some personal and professional factors. For example, younger teachers are more likely to be enthusiastic about their new teaching careers, whilst older teachers may experience boredom leading to increased exhaustion. Consistent with this hypothesis, one study reported that teachers who had taught for the fewest (0–5) years experienced the lowest burnout prevalence [54]. On the contrary, more experienced teachers were likely to have gained exposure, learnt students’ characteristics and classroom management skills and the necessary tools to help them prevent and address burnout. Additionally, teachers who lacked self-fulfilment may have been mostly younger and lacked personal accomplishments [47], leading to more burnout.

Significantly higher burnout scores, including for emotional exhaustion, depersonalization, and intellectual burnout were found among female teachers than among male teachers in some studies [51,52,53], whilst other studies reported that burnout was higher among male teachers. These results are contrary to findings reported among police officers, which indicated no significant difference in the levels of occupational burnout reported by male and female police officers [105]. Further studies are needed to investigate the contradictory gender differences in teachers’ burnout by different studies. In addition, research is needed on innovative gender-neutral ways of addressing burnout in teachers. Other structural factors, such as the number of children teachers have and class sizes which are associated with increased teacher burnout, require an increased investment in teachers and schools to address them. Governments providing teachers with affordable childcare and other supports for their own children, and building more schools to reduce the class sizes, may lead to a reduced burnout among teachers.

There is also a relationship between burnout and school or work-related factors. The subjects and grades taught and the medium of instruction all contribute to teachers’ burnout [7,51]. Teachers’ perceptions of the difficulty of a subject taught appears to determine their degree of burnout experienced; however, no particular subject seems to be the leading cause of burnout. High school teachers may perceive an increased workload in terms of the amount of time attributed to class preparation due to the difficulty of a subject taught. A cross-sectional study among nurses also found that role overload contributed to higher levels of emotional exhaustion [116] and this was also endorsed among healthcare managers where prolonged job strain resulted in burnout and an increased turnover intention [117]. This suggests there is a complex interaction between self-perception and burnout, which makes burnout in teachers a complex problem to address. Differences were also noted in the prevalence of burnout among teachers working in different countries [84]. For example, 58% of the variance in burnout in Cyprus could be explained by job satisfaction and anxiety, whereas 57.5% of the variance in burnout in Germany was explained by job satisfaction alone [84]. Different countries have different working conditions which may explain the differences in job satisfaction and associated burnout prevalence among teachers in different countries.

### 4.3. Effect of Resilience on Burnout

Resilience involves adapting well in the face of stress, difficulty, trauma, disaster, and threats. Resilient people use positive emotions to rebound and find positive meaning even in stressful circumstances [118]. Resilience had a significantinverse correlation with job burnout and turnover intention, and resilience could negatively predict job burnout [86]. Resilience was also reported to have an inverse association with burnout symptoms [119]; thus, increased resilience is linked to decreased burnout and, hence, the tendency for a teacher to remain in their job and thrive no matter what they encounter. Job burnout had a significant positive predictive effect and correlation with turnover intention, which suggests that the more severe the job burnout is, the higher the turnover intention [86]. Teachers require positive emotions and an increased resilience to remain in the profession and succeed without quitting. Conversely, among physicians, a survey indicated that the burnout prevalence was still significant even among the most resilient physicians; however, West et al. suggested that physicians exhibited higher levels of resilience than the general working population [119], including teachers. Additionally, resilience was also a significant predictor of depression and anxiety [88]; thus, the higher the resilience, the less likely teachers will experience depression or anxiety.

### 4.4. Socio-Demographic, School and Work-Related Factors as Determinants of Depression and Anxiety

Socio-demographic, school and work-related factors are all associated with both anxiety and depression [42,50,51,80]. This association is consistent with what was reported in a systematic review and meta-analysis by Ma et al., which suggested that teachers’ experiences of psychological issues were associated with various socio-demographic factors such as gender, institutional factors, teaching experience, and workload volume [100]. In this scoping review, conflicting results were found in relation to the association between teacher gender and depression. Whilst some studies reported that female teachers have higher depression levels than male teachers [42,51,70,79,81,82], other studies have reported no gender differences in teacher depression levels [53]. Contradictory results were also reported for the association between the age of teachers and depression, with some studies reporting higher depression levels in younger teachers [42] and others reporting higher depression in older teachers [51]. As discussed previously, it is likely that the use of different scales, coupled with organizational factors, contributed to these contradictory findings among the different studies. The findings also indicated that most female teachers who suffered from depression had been working for about 11 to 15 years [120].

A poor workplace environment has also been associated with increased anxiety and depressive symptoms [121] and school-related stress may transition to depressive symptoms among teachers [80,94]. As teachers’ workloads increase, their working hours will invariably increase, resulting in a rise in job demand and ultimately a surge in stress, leading to anxiety and depression. A systematic review reported similar findings where the main risk factors associated with anxiety and depression included job overload and job demands. [122]. The research also shows that teachers are not the only exception regarding experiencing a poor workplace environment which may lead to increased anxiety and depression [122,123]. Improving teachers’ workplace environments may, therefore, reduce the prevalence of anxiety and depression among teachers. Anxiety has also been linked to stressors relating to pupils and parents. For example, the possibility of a parental complaint increased anxiety scores [45]. Generally, parents want their children to succeed academically, which sometimes creates friction between teachers and parents. The underperformance of students or failure may be blamed on teachers or construed as the responsibility of schools and teachers [124], which may result in increased stress and subsequently anxiety and depression for teachers.

Social support was also reported to predict anxiety and depression symptoms, with high support levels indicating fewer symptoms related to anxiety and severe depression [121,125]; thus, teachers who perceived social support at school (e.g., the personnel relation dimension) expressed a lower stress level than those who did not [75]. According to Peele and Wolf 2020, anxiety and depressive symptoms increase for all teachers over the school year, and poor social support plays a significant role in the development of anxiety and depression symptoms [121]. Organizational policies that include the provision of adequate social support for teachers may, therefore, be a useful strategy to prevent and mitigate anxiety and depressive symptoms among teachers.

## 5. Limitations

The scoping review is not without limitations. This scoping review searched for articles in the English language only. Though every effort was made to identify all relevant studies for this review considering our eligibility criteria, we may have left out some relevant studies, particularly those published in other languages. Our search included six databases, yet the overall search strategy may have been biased toward health and sciences. Searching other bibliographic databases may have yielded additional published articles. Furthermore, different studies included in this scoping review used various screening tools and worldwide diagnostic classifications to determine stress, burnout, anxiety, and depression, leading to variations in the prevalence estimates. The scoping review included studies from 1974 till date; therefore, it is possible that the theoretical approaches to the concept of burnout may have changed. Notwithstanding these potential changes in the theoretical approaches to the concept of burnout, the burnout prevalence among teachers has appeared to have remained stable over the years. There was also no evaluation of the risk of bias for the included studies. Despite these limitations, this scoping review provides an excellent perspective on the prevalence and correlates of stress, burnout, anxiety and depression among teachers.

## 6. Conclusions 

Teachers’ psychological and mental health is of utmost importance as it indirectly affects the students they teach. The stress associated with the teaching profession can be linked to three major overlapping issues: burnout, anxiety, and depression, which have a myriad of effects, including an impact on teachers’ health, well-being, and productivity. A wide range of prevalences and correlates were reported for stress, burnout, anxiety, and depression. Differences in the severity were observed in different articles resulting in the diverse prevalence reported among the various studies. The differences in the measurement instruments creates critical knowledge gaps, making it difficult for researchers to make effective comparisons between the different studies. Future research should focus on addressing these research gaps arising from methodological issues, especially the use of different scales to allow for a meaningful comparison. Researchers, educators, and policy makers could benefit from an international consensus meeting and agree on common scales to be used when assessing stress, burnout, anxiety, and depression in teachers. Such an international consensus meeting can also help to streamline the definition of stress and can be used as a forum for addressing other methodological issues related to research and innovations involving elementary and high school teachers. Future research can also focus on exploring the gender differences in these psychological issues further, especially, defining the various subsets of gender being referred to and the specific prevalence in each case. In addition, the high prevalence of stress, burnout, anxiety, and depression reported particularly by several high-quality studies suggests that these psychological problems are widespread among teachers and deserves special attention both at the level of policy and practice.

This scoping review also highlights the risk factors associated with stress, burnout, anxiety, and depression. Identifying these risk factors is a significant step toward addressing these issues among teachers. Schools need to prioritize and promote interventions aimed at teachers’ personal wellbeing. Testing and implementing the interventions aiming to improve teachers’ well-being and ability to cope are important to address stress and burnout, with the expectation that this will prevent or reduce anxiety and depression. This may include school-based awareness and intervention programs to detect the early signs of teacher stress and burnout, or programs that incorporate meditation techniques or text-based support. Meditation techniques have been proposed to be effective in improving psychological distress, fatigue and burnout [126]. For example, mindfulness practice has been suggested as beneficial in coping with job-related stress, improving the sense of efficacy and reducing burnout in the teaching profession [127]. Interventions such as mobile text technology are an evidence-based, unique, and innovative way that offers a convenient, low cost and easily accessible form of delivering psychological interventions to the public with mental health problems [128,129,130]. Mobile text-based programs can be easily implemented at the school level to support teachers’ psychological needs. Future studies need to explore the development, implementation, monitoring, and evaluation of intervention programs for improving mental health outcomes among teachers. For instance, the Wellness4Teachers program which is planned for implementation in Alberta and Nova Scotia, Canada [34], is expected to provide evidence of effectiveness for the use of daily supportive text messaging to combat stress, burnout, anxiety, and depression among teachers. Finally, governments, school boards and policymakers need to collaborate with researchers on the design and implementation of measures to enhance teachers’ mental health, productivity (teaching) and quality of life.

## Figures and Tables

**Table 1:** Demographic, school and professional correlates of burnout, stress, anxiety and depression.

Variables	Burnout		Stress		Anxiety		Depression	
	Correlates	Citations	Correlates	Citations	Correlates	Citations	Correlates	Citations
**Demographic Correlates**								
Sex	✓	[40,47,51,52,53,54,55,63,67,68,72,73]	✓	[42,53,57,66,74,75,76,77,78]	✓	[42,79]	✓	[28,41,52,63,74,80]
Age	✓	[40,47,51,52,53,54,55,63,67,68,72,73]	✓	[42,53,57,66,74,75,76,77,78]	✓	[28,79]	✓	[28,42,51,70,81,82]
Gender	✓	[40,47,51,52,53,54,55,63,67,68,72,73]	✓	[42,53,57,66,74,75,76,77,78]	✓	[42,79]	✓	[28,42,51,70,81,82]
Marital Status	✓	[40,47,51,52,53,54,55,63,67,68,72,73]			✓	[66]		
Years taught/Teaching Experience	✓	[40,47,51,52,53,54,55,63,67,68,72,73]			✓	[40]	✓	[42]
Educational Level							✓	[42]
Family economics status and income	✓	[40]			✓	[40]	✓	[40]
Teachers’ weight	✓	[55]						
Spirituality			✓	[83]				
Number of children	✓	[63]						
Country of participant			✓	[39]				
School and professional correlates								
Work factors/job strain	✓	[18,42,67,84]	✓	[42,45,77,78]	✓	[42]	✓	[42,50,51,80]
Subjects/Level taught	✓	[7,51,72]	✓	[75,78,85]	✓	[51,65]	✓	[42,50,51,80]
**School Climate/Organizational Justice**							✓	[70]
Job Satisfaction/Absenteeism			✓	[39,43,82]	✓	[51,65]	✓	[42,50,51,80,81]
Student type/Behavior			✓	[45,77]			✓	[42,50,51,80]
Teaching special needs			✓	[78]			✓	[50]
Lack of students’ Progress			✓	[78,85]				
Violence/Verbal Abuse from Students							✓	[82]
Dealing with parent			✓	[45]				
Class Management			✓	[45]				
High job demands and workload	✓	[73,86]	✓	[42,53,57,74,75,76,77,78]			✓	[73,87]
Resilience/Class size	✓	[40,86]	✓	[78,85,88]	✓	[40]	✓	[40]
Role conflict, Role ambiguity Role Clarity	✓	[3,89]	✓	[89]			✓	[87]
Collective efficacy, school climate, and organizational justice	✓	[70]					✓	[70,90]
Student motivation and time pressure	✓	[18]						
School type/Income	✓	[40,70]	✓	[82]				
Interpersonal conflict and organizational constraints	✓					[73]		
Job seniority	✓	[73]						
High sense of coherence among colleagues			✓	[91]			✓	[91]
Student Attendance							✓	[81]
**Social and other correlates**								
Dysfunctional attitudes, ruminative responses, and pessimistic attributions.	✓	[92]					✓	[92]
Exercise	✓	[40]					✓	[61]
Relationship quality	✓	[40]					✓	[40]
Presenteeism							✓	[81]
Absenteeism					✓	[65]	✓	[31]
Non-restorative sleep							✓	[80]
Effort-reward imbalance							✓	[42,50,51,80]
Quality of life							✓	[31]
Psychological distress			✓	[74]				
Communication					✓	[58]		
Overcommitment	✓	[50]	✓	[85]				
Flourishing/Languishing							✓	[59]
Being a Refugee			✓	[57]				
Humiliation/Discrimination/mobbing			✓	[93]				
Self-care			✓	[57]	✓	[57]	✓	[57]
Neuroticism	✓	[67]						
Internet addiction	✓	[48]					✓	[48]
Drinking/Smoking	✓	[40]					✓	[61]
Confidence levels	✓	[54,69]						
Motivation to quit	✓	[18]						
General lifestyle	✓	[54,69]						

## Data Availability

Not applicable.

## Appendix A

**Table A1:** Summary of studies with prevalence and correlates of Burnout/Stress.

Authors/Year	Country	Study Design	Sample/Population Size (Response Rate %)	Teachers/Age Range	Scales Used	Key Findings	
						Correlates of Burnout/Stress	Prevalence of Burnout/Stress
Okwaraji et al., 2015	[47] Nigeria	Cross-sectional	SS = 432	Secondary 26–48 years	Maslach burnout inventory, The General health questionnaire (GHQ-12) and the Generic job satisfaction scale	DP: gender, marital status Reduced PA: age, gender, marital status.	40% emotional exhaustion EE 39.4% for DP 36.8% for reduced PA.
Kidger et al., 2016 [81]	UK	Cross-sectional	555/708/ (78.4%)	Secondary	Warwick Edinburgh Mental Wellbeing Scale-WEMWBS)	Stress at work: change in school governance.	Not Mentioned.
Bianchi et al., 2015 [99]	France	Survey	SS = 627	Primary/Secondary	Maslach Burnout Inventory (MBI)	Burnout symptoms at time 1 (Tl) did not predict depressive symptoms at time 2 (T2).	Time 1 43%, mild burnout 49% moderate burnout, 8% severe burnout.
Ramberg et al., 2021 [91]	Sweden	Cross-sectional	Year 2014/16 3948/7147 (55.2%) SS Final = 2732	Teachers	Stockholm Teacher Survey. The (Questionnaire)	Perceived stress: high job strain, high SOC. Stress: psychological demands at work. High SOC was linked with lower levels of stress and depressed mood. Variation of 4.8% for perceived stress and 2.1% for depressed mood.	Not mentioned.
Shukla et al., 2008 [7]	India	Survey	SS = 320	Secondary	Maslach Burnout Inventory	Lack of PA: subject taught. Science teachers’ higher burnout than arts teachers. More burnout cases in English medium teachers than Hindi medium. Burnout: gender.	EE: 56.56% low burnout, 19.68% average, 23.75% high. DP: 20% high burnout, 16.56% average, and 63.43% low. Lack of PA: 28.43% high burnout. 13.43% average, and 58.12% low. Lack of PA: 28.43% 11.88% high burnout level in all 3 dimensions, 2.81% average burnout on all 3 sub-scales and 40% low burnout level in all dimensions. Burnout of SCIS teachers 26.26%, (AS, 13.76%. EE: 22.5% SCIS and 25% AS teachers’ high burnout category, 21.88% SCIS and 17.5% AS teachers’ average burnout level, 55.62% SCIS and 57.5% AS teachers’ low burnout. Approximately 56–64% in all dimensions of the sample is showing low burnout levels.
Pohl et al., 2022 [48]	Hungary	Cross-sectional	1817/2500 (72.7%)	High school/18–65	Maslach Burnout Inventory.	Severe burnout, EE and DP: Internet addiction Internet addiction was associated with severe burnout (10.5 vs. 2.7%, *p* < 0.001), moderate (36.8 vs. 1.7%, *p* < 0.001), and severe (6.3 vs. 0.1%, *p* < 0.001).	26.0% mild, 70.9% moderate, and 3.1% severe burnout.
Papastylianou et al., 2009 [3]	Greece	Cross-sectional	562/985 (57.1%)	Primary/30–45	Maslach and Jackson, MBI: Maslach Burnout Inventory.	EE: depressed affect, positive affect, degree of role clarity, role conflict and role ambiguity.	EE: 25.09%, PA 14.27% and DP: 8.65%.
Hadi et al., 2009 [76]	Malaysia	Cross-sectional	565/580 (97.4%)	Female/male Mean age 40.5	Depression, Anxiety and Stress Scale (DASS 21) and Job Content Questionnaire (JCQ).	Stress: age, duration of work and psychological job demands.	34.0% stress, 17.4% of teachers experienced mild stress.
Ratanasiripong et al., 2021 [40]	Thailand	Cross-sectional	SS = 267	Primary/secondary 44.4	The Maslach Burnout Inventory for Educators Survey, Thai version (MBI-ES).	Stress: marital status negative relation with stress., Family economics status, gender, sleep and resilience. Burnout (EE): relationship quality and age. DD: relationship quality and drinking. PA: resilience and number of teaching hours.	6.0% had severe to extremely severe stress.
Szigeti et al., 2017 [50]	Hungary	Cross-sectional	SS = 211	Primary/secondary 42.8	Hungarian version of the MBI–ES	General burnout/EE: overcommitment	General burnout 58%, 13% for EE 11% for DP, and 17% for PA.
Hodge et al., 1974 [72]	Wales, England	Cross-sectional	107/145 (75%)	Secondary, 33 mean	Maslach Burnout Inventory (MBI) and General Health Questionnaire (GHQ-60).	EE: difficulty of subject taught and satisfaction, age. 58% of music teachers thought subject was the most difficult subject to teach, 29% of mathematics teachers.	Music teachers have significantly higher EE and DP (high burnt) scores than mathematics teachers. Music teachers.
Baka 2015 [73]	Poland	Cross-sectional	316/400/ (79%)	Primary/secondary 22–60	The Oldenburg Burnout Inventory.	Job burnout: age and job seniority, work hours, job demands. Job burnout decreases along with age and job seniority. Increased work hours were accompanied by job demands, general job burnout, depression and physical symptoms.	Not mentioned.
Othman et al., 2019 [131]	Malaysia	Cross-sectional	SS = 356	Secondary <20->/= 50	Malay Depression Anxiety Stress Scales (DASS).	Stress; gender, educational status, teaching experience, marital status.	32.3% stress symptoms 25.3% were mild to moderate. 7.0% severe to the extremely severe stress. Female stress 32.7%, Indian/other ethnic 50.6%, lowest educational status 46.1%, longest teaching experience (34.6%), lowest income (33.9%), marriage duration 11–20 years (37.3%), 1–3 children (35.5%),
Skaalvik et al., 2020 [18]	Norway	Longitudinal	SS = 262	High school	Maslach Burnout Inventory-Educators Survey.	EE: time pressure. Cynicism: low student motivation. Self-perceived accomplishment: autonomy and low student motivation. Burnout: motivation to quit, job satisfaction.	Not mentioned
Li et al., 2020 [55]	China	Cross-sectional	1741/1795 (97%)	Kindergartens/preschool 18–48	Chinese version Maslach Burnout Inventory and the Perceived Stress Scale-14.	Burnout rate: overweight/obesity, type of school, income satisfaction, depression. Burnout: age, higher perceived stress levels, shorter years of teaching. Perceived stress (*p* < 0.001, OR = 1.15, 95%CI: 1.13–1.18).	Burnout was 53.2%. 53.0% (851/1607) in female subjects and 56.0% (75/134) in male subjects.
Gosnell et al., 2021 [57]	Malaysia	Cross-sectional	123/400(31%)	Primary/secondary	Depression Anxiety Stress Scales-21 self-care strategy questionnaire was adapted from a self-care scale in the Mental Health Handbook.	Stress: self-care. The association was moderated by age. Among refugee teachers, women were more stressed than men. Stress: negative correlation with age. Younger teachers experienced higher rates of stress than older teachers.	Refugee teachers 8.3% in the severe or extremely severe stress levels clinical ranges.
Capone et al., 2020 [59]	Italy		SS = 285	High school 29–65	Burnout Inventory- General Survey (MBI).	EE, and DP: flourishing participants languishing teachers.	22.1% for EE and 9.5% for DP.
Chan et al., 2002 [74]	China	Cross-sectional	SS = 83	Secondary 22–42	The shortened 20-item Teacher Stressor Scale (TSS). e 20-item Chinese shortened version of the General Health Questionnaire (GHQ-20).	Stress: psychological distress. Gender, age. Self-efficacy: psychological distress, social support.	Not mentioned.
Zhang et al., 2014 [52]	China	Survey	SS = 590	Primary/secondary 34 ± 8.11	Chinese Maslach Burnout Inventory.	Reduced PA and intellectual burnout: somatization EE, DP, and intellectual burnout: gender. Burnout: gender, level of mental health. EE, DP: best predictor anxiety.	EE accounted for 92.8% of the burnout cases, DP for 92.9%, reduced PA for 89.9%, and intellectual burnout for 95.0%). Burnout is more severe in female teachers than in male teachers.
Vladut, et al., 2011 [69]	Romania	Cross-sectional	SS = 177	Primary/secondary/High 22–64	Maslach Burnout Inventory (MBI). Teachers’ Sense of Efficacy Scale.	Burnout: rural or urban teaching, self-acceptance, classroom management, work-conditions and confidence.	49.6% above moderate or severe EE 28.7% on DP 54.1% on inefficacy.
Liu et al., 2021 [86]	China	Cross-sectional	449/500 (89.8%)	High 36.70	Maslach Burnout Inventory (MBI).	Job burnout: turnover intention; resilience has negative correlation. EE was the most predictive factor for turnover intention with an explanatory variance of 29.2%, followed by DP with an explanatory variance of 1.9% Lest is low PA with 1.5%.	Not mentioned.
Fimian et al., 1983 [44]	US	Survey	365/800(47%)	Special education	Teacher Stress Inventory (TSI) Survey. Sources of Stress (25 items); Emotional and Behavioral Manifestations of Stress (24 items); Physiological Manifestations of Stress (16 items).	Stress: lack of time to spend with individual pupils, teaching. Special needs, or mixed ability students. Increased workload, feeling isolated, and frustrated because of poor administration attitudes and behaviors.	87.1% moderately-to-very stressful. (45.6%) much-to-very-much stress. 15.9% (58/365) identified as low-stress, (68.4% (250/365) as moderate-stress, and 15.6% (57/365) as high-stress teachers.
Katsantonis 2020 [39]	* 15 Countries.	Survey	SS = 51,782	Primary	Self-efficacy is domain-specific and three scales reflect the self-efficacy. 5 items scale was designed by OECD (2019) to measure factors that cause workload stress.	Workload stress: self-efficacy in instruction, student-behavior, workplace well-being, work satisfaction. Stress: perceived disciplinary climate. School climate negative effect. Increase work satisfaction results in perceived less stress. 16% (organizational constraints as a predictor of depression).	Japanese participants had greater levels of workload stress than Korean participants. Participants from Belgium perceived greater workload stress.
Ratanasiripong et al., 2020 [88]	Japan	Cross-sectional	174/200 (87%)	Primary/secondary 41.65	Japanese version of depression, Anxiety, and Stress scale (DASS-42). Japanese version of the Connor–Davidson Resilience Scale (CD-RISC). Japanese version of the Rosenberg Self-Esteem Scale (RSE).	Stress: resiliency and self-esteem. Strength Higher self-esteem and resilience were significantly correlated to less stress.	Not mentioned.
Jurado et al., 2005 [82]	Spain	Cross-sectional	496/602/ (82.7%)	Primary/secondary (women, 45.3 ± 9.8; men, 44.7 ± 9.7).	Spanish version of Epidemiologic Studies Depression scale (CES-D).	Job stress: negative correlation with job satisfaction, desire to change job and appraisal by others. Teachers wishing to change jobs (25%; significantly higher score on job stress but low on job satisfaction and appraisal by others.	
Bianchi et al., 2021 [132]	France Spain Switzerland	Survey	France (*N* = 4395), Spain (*N* = 611), and Switzerland (*N* = 514)	Schoolteachers	Maslach Burnout Inventory for Educators. Job strain was measured with a shortened version of the Effort-Reward Imbalance Questionnaire.	Burnout: neuroticism prediction (28–34%), job strain (10–12%), skill development, security in daily life, and work–non-work conflict (about 15–18%), sex, age, unreasonable work tasks, workhours, job autonomy, sentimental accomplishment, leisure activities, personal life support.	Not mentioned.
Bianchi et al., 2014 [60]	France	Analytical	SS = 5575	School teachers 41 years;	Maslach Burnout Inventory. Depression was measured with the 9-item depression scale of the Patient Health Questionnaire (PHQ-9).	EE: Strongly associated with depression than with DP and reduced PA.	No-burnout 13% (750) participants.
Hammen et al., 1982 [43]	US	Cross-sectional	SS = 75	Secondary	DASS-21scale. Bruno’s Teacher stress Inventory	Stress: depressive symptomatology, days off work, school-related factors.	76% moderate or greater stress 20% level of stress was “almost unbearable.”
Méndez et al., 2020 [25]	Spain	Cross-sectional	210/300 (70%)	30 to 65	Maslach burnout inventory.	Burnout: correlates with EE, PA and DP resulting in three burnout profiles (high burnout); (moderate burnout) and (low burnout). Burnout: depressive symptomatology. The higher the burnout the greater the depressive symptomatology	33.3% high burnout 39.1% low burnout and 27.6% moderate burnout.
Jepson et al., 2006 [85]	UK	Cross-sectional	95/159 (60%)	Primary/secondary	Perceived Stress Scale (PSS). 10 scale item, occupational commitment 6 scale item.	Work-related stress, strongest predictor and negative relationship, was occupational commitment, achievement striving experience, level taught. Educational level taught. Occupational commitment increases, perceived stress decreases.	Significantly higher levels of perceived stress were reported from primary school teachers than secondary school. Higher achievement striving experience have higher levels of perceived stress.
Al-Gelban 2008 [96]	Saudi Arabia	Cross-sectional	195/189 (96.9%)	Male 28–57	Depression, Anxiety and stress DASS-42 scale.	Depression, anxiety and stress were strongly positively and significantly correlated.	31% had stress.
Lee et al., 2020 [120]	Malaysia	Cross-sectional	SS = 150	Secondary/primary	DASS-21 inventory.	Stress: number of years working. Majority of teachers with stress: either severe and extremely severe level are those working for 11 to 15 years.	10.7% stress.
Bounds et al., 2018 [111]	US	Survey	108/117 (92%)	Primary/secondary 42	Teacher Stress Inventory (TSI).	Stress: violence against, urban, suburban, and rural setting.	Urban teachers had the highest levels of stress from violence rather than suburban teachers.
Pressley et al., 2021 [56]	US	Survey	SS = 329	Elementary	The COVID Anxiety Scale. A teacher burnout subscale of stress.	Stress: anxiety factors in pandemic situations.	Not mentioned.
Yaman 2015 [93]	Turkey	Survey	SS = 436	Elementary/branch 35.2	Mobbing Scale and the Stress subscale of the Depression Anxiety Stress Scale. Turkish version of the Stress Subscale of DASS.	Stress: predicted positively by humiliation, discrimination, communication barriers, and mobbing scores.	Increment in mobbing will increase stress.
Cook et al., 2019 [83]	US	Cross-sectional	180/105/58.5%	Middle 22 ± 37	Teacher Stress Inventory. The Daily Spiritual Experience Scale.	Stress: teacher spirituality. As teachers’ spirituality increases, their time-management stress and their work-related stress increase.	Not mentioned.
Okebukolal 1992 [75]	Nigeria	Survey	SS = 368	Science	The Occupational Stress Inventory for Science Teachers (OSIST).	Stress: school villages (personnel relation dimension) curriculum, facilities, student characteristics, administrative, and professional growth and self-satisfaction, subject taught, science budget. Science teachers in the rural schools mean stress score of 47.25 (SD = 4.89), urban schools mean stress score of 51.29 (SD = 6.95).	Urban teachers were found to be more stressed than those in rural areas. Female science teachers were more stressed than their male counterparts.
Klassen 2010 [77]	Canada	Survey	951/- (Approximately 75%)	Elementary/secondary	Teacher Stress Inventory. Collective Teacher Efficacy Belief Scale (CTEBS Job satisfaction was measured with a one-factor, three-item, 9-point Likert-type scale.	Stress: collective efficacy, student behavior, gender, workload, class size.	21.3% females rated the stress from workload “quite a bit” or “a great deal” of stress from workload factors. 13.4% of male teachers rated stress from workload at a mean of 7 or higher. More women (18.6%) than men (12.8%) reported feeling “quite a bit” or “a great deal” of stress from student behavior.
Proctor et al., 1992 [45]	UK	Survey	256 (93%)	Primary 39.68	Zigmond and Snaith’s 6 Hospital Anxiety and Depression (HAD) Scale and Moos and Insel’s7 Work Environment Scale (WES).	Stress: anxiety, work overload, time pressures, stressors relating to pupils and parents.	67% found teaching ‘considerably’ or ‘extremely’ stressful, 79 (32%) ‘slightly’ stressful and 2 (1%) ‘not at all’ stressful.
Akin 2019 [63]	Turkey	Mixed research method	460/3478 (13%)	Teachers	Turkish version of the Maslach and Jackson inventory.	DP: marital status. Reduced PA: number of children.	Not mentioned.
Chan 1998 [125]	Hong Kong	Cross-sectional	SS = 415	Secondary 21–61	Teacher stressor scale and the General Health Questionnaire.	Stress: high support—less anxiety symptoms, psychological symptoms.	37.3% psychiatry morbidity.
Adeniyi et al., 2010 [78]	Nigeria	Cross-sectional	SS = 50	Special Needs	Job Stress Inventory.	Stress: marital status, teaching special needs, lack of pupils’ progress in class work/academic achievement, societal attitudes/respect heavy workload and lack of help/assistance, degree and nature of disabilities of the special need children.	Not mentioned.
Beer et al., 1992 [53]	US	Cross-sectional	86/92(93%)	Grade and high school	Beck’s Depression Scale, the Coopersmith Self-esteem Inventory—Adult Form, Stress Profile for Teachers, and the Staff Burnout Scale.	Burnout and stress: gender, level taught-high/grade school. Grade school teachers experienced more burnout than high school teachers.	Burnout scores higher for female high school teachers than for both male and female grade school teachers. Scores on stress were higher for male high school teachers than for both female high school teachers and male grade school teachers.
Liu et al., 2021 [98]	China	Cross-sectional	907/1004 (90.3%)	Primary and secondary 20 ≥ 50	Generic Scale of Phubbing, the Maslach Burnout Inventory—General Survey, Ruminative Response Scale, and the Center for Epidemiological Studies Depression Scale.	Job burnout: phubbing significant positive effect on job burnout, depression. The relation between job burnout and depression were moderated by rumination.	Not mentioned.
Shin et al., 2013 [95]	Korea	Survey	SS = 499	Middle and high school	Maslach Burnout Inventory–Educator Survey Center for Epidemiological Studies Depression Scale.	Burnout: depression; baseline status of depression. Teacher’s burnout leads to subsequent depression symptoms, not vice versa.	Not mentioned.
Genoud et al., 2021 [41]	Switzerland	Cross- sectional	SS = 470	Secondary 24–63	Maslach’s burnout scale version validated by Dion and Tessier twenty-seven items French; Depression Anxiety Stress Scales (DASS).	Burnout: negative affectivity (tendency to feel depression, anxiety, or stress), personal fulfillment. Greater tendency to feel depressed result in teachers experiencing a lower level of personal accomplishment.	Two-thirds of the sample (N = 308) 66% of teachers below average for the three dimensions (stress, depression, and anxiety).
Steinhardt et al., 2011 [68]	US	Cross-sectional	/267 (26%)	High/Elementary/middle Mean 45	Maslach Burnout Inventory-Educators Survey (MBI-ES) Modified version of the Teacher Stress Inventory.	Burnout: gender, experienced. Stress: depressive symptoms. Females reported greater chronic work stress and emotional exhaustion. Total effect of stress on depressive symptoms, taking together the direct and indirect effects via burnout, accounted for 43% of the total variance.	Increased stress leads to increased burned out.
Pressley 2021 [58]	US	Survey	SS = 359	Primary/secondary	Teacher burnout scales.	Burnout-stress: COVID-19 anxiety, current teaching anxiety, anxiety communicating with parents, and administrative support.	High level of average teacher burnout stress score of 24.85.
Schonfeld et al., 2016 [64]	US	Survey	SS + 1386	School teachers mean = 43	The Shirom-Melamed Burnout Measure, Depression module of the Patient Health Questionnaire.	Burnout and depressive symptoms were strongly correlated. Burnout and depressive symptoms: stressful life events, job adversity, and workplace support. Burnout: anxiety. 86% of the teachers identified as burned out met criteria for a provisional diagnosis of depression. Fewer than 1% in the no-burnout group.	Not mentioned
Bianchi et al., 2016 [92]	New Zealand	Cross-sectional	SS = 184	School teachers Mean 43	Shirom–Melamed Burnout Measure (SMBM) Depression was assessed with the PHQ-9.	Burnout: strongly correlation. Depressive symptoms, moderately correlated with dysfunctional attitudes, ruminative responses, and pessimistic attributions.	Depression “low burnout-depression”, (*n* = 56; 30%), “Medium burnout-depression” (*n* = 82; 45%), “High burnout-depression” (*n* = 46; 25%). (About 8%) reported burnout symptoms at high frequencies and were identified as clinically depressed.
Desouky and Allam 2017 [28]	Egypt	Cross-sectional	SS = 568	High 39.4 ± 8.7	Arabic version of the Occupational Stress Index (OSI), the Arabic validated versions of Taylor manifest anxiety scale and the Beck Depression Inventory.	OS: Anxiety and depression scores, age, gender, higher qualifications and higher workload. OS, anxiety and depression scores were significantly higher among teachers with an age more than 40 years, female teachers, primary school teachers, higher teaching experience.	OS, anxiety and depression, respectively. 100%, 67.5% and 23.2%, Private schools show a significantly higher prevalence of moderate and severe OS compared to governmental schools (31.6% and 68.4% vs. 22.4% and 67.1%).
Jones-Rincon et al., 2019 [65]	US	Cross-sectional	3003/3361(89%)	Elementary, middle/junior high or high	Patient Health Questionnaire. Job satisfaction was measured with 10 items.	Perceived stress levels: anxiety disorder. Teachers with anxiety disorder reported having higher perceived stress levels.	Not mentioned.
Kinnunen et al., 1994 [51]	Finland	Survey	1012/1308/ (77%)	High/vocational/special/Physical/secondary 45–59	Maslach and Jackson’s inventory.	EE: gender. Poor work ability. Women exhibit higher scores for EE.	Not Mentioned
Martínez et al., 2020 [46]	Spain	Random Sampling	215/300 (71.7%)	Primary 30 to 65 years *M* = 44.89	The Maslach Burnout Inventory (MBI), Zung Self-Rating Depression Scale (SDS), Coping with Stress Questionnaire.	Burnout: depressive symptomatology, and quality of interpersonal relationships.	48.37% low levels of EE, 25.12% high levels of PA, (b) high levels of EE and DP, and (c) 26.51% low levels of DE and PA.
Capone et al., 2019 [70]	Italy	Cross-sectional	SS = 609	High school, middle school, elementary and primary school. 27 to 65, mean = 48.35	The Center for Epidemiologic Studies Depression Scale (Italian version. The Italian version of the Maslach Burnout Inventory-General Scale. The Teacher Self-Efficacy Scale.	Burnout: collective efficacy, school climate, and organizational justice and relationship. EE and cynicism functioned as significant mediators between the three predictors (opportunities, organizational relationships, and organizational justice) and depression.	Not mentioned.
Aydogan 2009 [84]	Turkey N = 83 Germany N = 78 Cyprus N = 74	Cross-sectional	255/306 (83%)	High M = 38 ± 6.96, 37.9 ± 6.74, 45.8 ± 10.42	Shirom–Melamed Burnout Measure. Turkish version of Minnesota Job satisfaction scale.	Burnout: country working, job satisfaction, depression. Cyprus teachers 57% of the variance in burnout explained by depression. 58% of the variance in burnout explained by job satisfaction and anxiety. Germany 575% variance in burnout explained by job satisfaction.	Not mentioned.
Belcastro et al., 1983 [49]	US	Cross-sectional	428/359 (84%)	Public	The Maslach Burnout Inventory and the Teacher Somatic Complaints and Illness Inventory.	burned-out: somatic complaints	More than 11% burned out. 246 (68.5%) not burned-out.
Capel 1992 [89]	UK	Cross-sectional	640/405/63.3%	Middle, upper, high school	The Maslach Burnout Inventory. The Taylor Manifest.	Stress and burnout: role conflict, and role ambiguity, High anxiety. Highest stress level: high workload demands after-school time, lack of recognition for extra work, too much paperwork. Students’ behavior. Burnout: anxiety.	Not mentioned.
Ptacek et al., 2019 [54]	Czech Republic	Cross-sectional	SS = 2394	Primary 18–72	Questionnaire survey: anamnestic part and Standardized questionnaires: SVF 78, SMBM, ENRICHD SSI, BDI II, USE.	Burnout: length of teaching/employment, healthy lifestyle. Cognitive burnout: age and length of teaching employment. Those with healthy lifestyle (work–life balance) have significantly lower burnout rates. Males–higher emotional burnout, females–higher physical burnout rates).	18.3% of participants felt definitely threatened by burnout syndrome, 34.9% may be, 9.9% definitely not threatened by burnout syndrome. Long-term stress 21.8%, compared to the (7.5%) do not experience long-term stress.

* Katsantonis 2020 (15 countries)—Japan and Korea form the East-Asian model. France and Spain form the Latin model. Denmark and Sweden form the Northern model. Australia and the United Kingdom represent the Anglo-Saxon model and finally, Belgium and the Netherlands form the Germanic model. Sample Size: SS; Emotional Exhaustion: EE; Personal Accomplishment: PA; Depersonalization: DP; Occupational Stress: OS; Sense of Coherence: SOC; Science Stream: SCIS; Art Stream: AS.

**Table A2:** Summary of studies with prevalence and correlates of Depression/Anxiety.

Authors/Year	Country	Study Design	Sample Size/Population Size (Response Rate)	Teachers/Age Range	Scales Used	Key Findings	
						Correlates of Depression/Anxiety	Prevalence of Depression/Anxiety
Jurado et al., 2005 [82]	Spain	Cross-sectional	498/602/ (82.7%)	Primary/secondary (women, 45.3 ± 9.8; men, 44.7 ± 9.7).	Spanish version of Epidemiologic Studies Depression scale (CES-D).	Depressive symptoms: female gender, age, low job satisfaction, high job stress, desire to change jobs, working at a public school, personality dimensions of harm avoidance (high), novelty seeking (high) and verbal insults from pupils.	Depressive symptoms 35.3% of the teachers.
Al-Gelban 2008 [96]	Saudi Arabia.	Cross-sectional	189/195 (96.9)	Male 28–57	Depression, Anxiety and stress DASS-42 scale.	Depression, anxiety, and stress were strongly, positively, and significantly correlated.	25% percent had depression 43% had anxiety.
Fimian et al., 1983 [44]	US	Survey	365/800 (47%)	Special education	Emotional and Behavioral Manifestations of Stress (24 items); and Physiological Manifestations of Stress (16 items).	Depressed/anxious: teaching special needs.	Not mentioned.
Lee et al., 2020 [120]	Malaysia	Cross-sectional	SS = 150	Female primary/secondary	DASS-21 inventory.	Depression: gender, years of work. Female teachers who suffered depression are those who have been working about 11–15 years.	15.3% depression; 30.7% anxiety.
Ratanasiripong et al., 2020 [88]	Japan	Cross-sectional	174/200 (87%)	Primary/secondary 41.65	Japanese version of depression, Anxiety, and Stress scale (DASS-42. Japanese version of the Connor-Davidson Resilience Scale (CD-RISC). Japanese version of the Rosenberg Self-Esteem Scale (RSE).	Depression and anxiety: resiliency and self-esteem, grade taught. Strength significantly predicted anxiety.	Anxiety in secondary school teachers significantly lower than elementary school teachers.
Schonfeld 1992 [90]	New York, US	Longitudinal	SS = 255	Women 27	Center for Epidemiologic Studies– Depression Scale (CES-D).	Depressive symptoms: work-environment, job satisfaction. Whites but not among principally Black and Hispanic subsample, motivation has negative affectivity.	Not mentioned.
Vladut, et al., 2011 [69]	Romania	Cross-sectional	SS = 177	Primary/secondary/high	The Depression, Anxiety and Stress Scale.	Anxiety/depression: burnout dimensions, demographic variables, mismatches between work-conditions gender, perception of reward and community.	Higher levels of emotional exhaustion. EE or DP and PA had significantly higher levels of depression, anxiety, and stress.
Bianchi et al., 2014 [60]	France	Analytical	SS = 5575	Teacher, mean 41	Depression was measured with the 9-item depression scale of the Patient Health Questionnaire (PHQ-9).	Depression: burnout:	90% of the teachers identified as burned out met diagnostic criteria for depression, mainly major depression (85%). 3% (*n* = 19) of the no-burnout group were identified as depressed, mainly minor depression or depression not otherwise specified (2%).
Hammen et al., 1982 [10]	US	Cross-sectional	SS = 75	Secondary	The Center for Epidemiological Studies-Depression (CES-D) scale.	Depressive symptomatology: stress, stress-related, cognitions regarding the consequences of the stressful circumstances, days off work.	8% reported major depression. 12% teachers met criteria for possible minor depression. 20% debilitating array of symptoms approximating a clinically significant depression syndrome.
Baka 2015 [73]	Poland	Survey	316/400 (79%)	Elementary/secondary 22–60	Depression (the Beck Hopelessness Scale).	Depression: 16% high organizational constraints predict depression. Interpersonal conflict, organizational constraints and 2% workload predicts depression.	Not mentioned.
Lee et al., 2020 [120]	Malaysia	Cross-sectional	SS = 150	female primary/secondary	DASS-21 inventory.	Depression: gender, years of work. Female teachers who suffered depression are those who have been working about 11 -15 years.	15.3% depression; 30.7% anxiety.
Pressley et al., 2021 [56]	US	Survey	SS = 329	Elementary	The COVID Anxiety Scale. A teacher burnout subscale of stress.	Anxiety: stress and communication within the school, and with parents, providing instruction in a virtual environment. Anxiety: COVID-19 pandemic. online teaching was positively related to anxiety in communications.	56.2% no change in anxiety. 38.9% of participants reported reduced anxiety, 4.9% of teachers felt more anxiety than their baseline at the 1st week of school. Almost 40% had a decrease in anxiety during the 1st month of the 2020–2021 school year.
Besse et al., 2015 [31]	US	Survey single-stage sample cluster	3003/3361 (89%)	Elementary, middle, or high school, mean = 43.9 years	Occupational health survey and Patient Health Questionnaire.	MDD: Hispanic, divorced, years of experience, taught at elementary level, low job satisfaction and higher absenteeism and increased likelihood of leaving the profession, perceived stress, anxiety.	Teachers with MDD had higher levels of perceived stress, anxiety.
Peele et al., 2020 [121]	Ghana	Randomized control trial	SS = 444	Kindergarten	Goldberg Anxiety and Depression Questionnaire.	Anxiety and depressive symptoms: poor workplace environment, social support, lack of parental support was associated with more anxiety (b = 0.12, *p* = 0.002), new to the local community. Depressive symptom: household food insecurity.	Poor workplace environment led to increased anxiety and depressive symptoms.
Beer and Beer 1992 [53]	US	Survey	86/92 (93)	Grade and high school	Beck’s Depression Scale, the Coopersmith Self-esteem Inventory—Adult Form, Stress Profile for Teachers, and the Staff Burnout Scale.	Depression: self-esteem, negative association. Teachers in an institutional setting, there is no significant difference for teaching level or sex on depression.	Not mentioned.
Proctor et al., 1992 [45]	UK	Survey	256 (93%)	Primary 39.68	Zigmond and Snaith’s 6 Hospital Anxiety and Depression (HAD) Scale and Moos and Insel’s7 Work Environment Scale (WES).	Anxiety/depression: stressors intrinsic to teaching and related to organizational factors within schools, ensuring pupil progress, work overload, time pressures, role conflict.	79% low or normal level of depression. 44 (17%) borderline scores and 10 (4%) clinical depression. Anxiety: 92 (36%) had normal scores and 67 (26%) borderline, 97 (38%) scored at a clinical level.
Liu et al., 2021 [98]	China.	Survey convenient sampling method	907/1004 (90.3%)	Primary and secondary 20 ± 50	Generic Scale of Phubbing, Ruminative Response Scale, and the Center for Epidemiological Studies Depression Scale.	Depression: phubbing. Combination of phubbing and rumination had no significant effect on depression.	Not mentioned.
Shin et al., 2013 [95]	Korea	Survey	SS = 499	Middle and high school	Maslach Burnout Inventory–Educator Survey Center for Epidemiological Studies Depression Scale.	Depression: burnout. Positive relationship between baseline status of teacher burnout and depression.	Not mentioned.
Genoud and Waroux 2021 [41]	Switzerland	Cross-sectional	SS = 470	Secondary 24–63	French: Depression Anxiety Stress Scales (DASS).	Anxious profile: emotional exhaustion. Depressive profile: sense of personal accomplishment, no negative affective trait.	66% (two-thirds) (N = 308) below average for the three dimensions (depression, anxiety, and stress).
Pohl et al., 2022 [48]	Hungary	Cross-sectional	1817//2500 (72.7%)	High 18–65	Beck Depression Inventory (BDI-SF). Problematic Internet Use Questionnaire.	Depression: internet addiction.	No depression 37.1% (673/1817), 58.9% (1070/1817) had mild, 3.5% (65/1817) had moderate and 0.6% (9/1817) had severe depression.
Steinhardt et al., 2011 [68]	US	Cross-sectional	/267 (26%)	High/elementary/middle, mean 45	The Center for Epidemiological Studies Depression Scale (CES-D).	Depressive symptoms: EE. Positive relationships with DP and reduced PA. Chronic work stress, experienced.	High school teachers reported greater depressive symptoms.
Pressley 2021 [58]	US	Survey	359	Primary/secondary	COVID Anxiety Scale.	Anxiety: stress, COVID-19, communicating with parents, administrative support, providing instruction in a virtual environment. Anxiety about online teaching was positively related to anxiety in communications.	Virtual instruction teachers have the most increase in anxiety.
Ratanasiripong et al., 2020 [88]	Japan	Cross-sectional	174/200 (87%)	Primary/secondary 41.65	Japanese version of depression, Anxiety, and Stress scale (DASS-42). Japanese version of the Connor-Davidson Resilience Scale (CD-RISC). Japanese version of the Rosenberg Self-Esteem Scale (RSE).	Resilience and self-esteem significantly predicted depression and anxiety.	Not mentioned.
Ptacek et al., 2019 [54]	Czech Republic	Survey	SS = 2394	Primary 18–72	Beck Depression Inventory II (BDI II).	Depression: burnout. There is a strong and significant correlation between burnout and depressive symptomatology.	15.2% mild to severe depression.
Bianchi et al., 2016 [92]	New Zealand	Cross-sectional	SS = 184	School teacher, mean 43	Depression was assessed with the PHQ-9.	Depressive symptoms: burnout, dysfunctional attitudes, ruminative responses, and pessimistic attributions.	Depression” low burnout-depression,” (*n* = 56; 30%), “medium burnout-depression” (*n* = 82; 45%), and “high burnout-depression” (*n* = 46; 25%). 14/184 (about 8%) reported.
Mahan et al., 2010 [71]	US	Cross-sectional	168/756 (23.9%)	High, mean 42.6	Ongoing Stressor Scale (OSS) and the Episodic Stressor Scale (ESS), the Co-worker and Supervisor Contents of Communication Scales (COCS), the State Anxiety inventory (S-Anxiety), and the Center for Epidemiological Studies Depression Scale (CES-D).	Anxiety and depression: ongoing and episodic stressors and support, 28% (adjusted 25%) of the variability in anxiety and 27% (adjusted 24%) of the variability in depression. Co-worker support had an inverse relationship to anxiety and depression, work environment stressor.	Higher levels of ongoing stressors, leads to higher levels of anxiety and depression, higher levels of co-worker support related to lower levels of anxiety and depression.
Desouky et al., 2017 [28]	Egypt	Cros-sectional	SS = 568	High	Arabic version of the Occupational Stress Index (OSI), the Arabic validated versions of Taylor manifest anxiety scale and the Beck Depression Inventory.	Anxiety and depression: occupational stress, OS), age, female teachers, primary school teachers, higher teaching experience, higher qualifications and higher workload.	OS anxiety and depression (100%, 67.5% and 23.2%), respectively. Mild, moderate and severe depressive symptoms among teachers was (19.7%, 2.8% and 0.7%), respectively, and little, mild, severe and very severe anxiety was (17.6%, 23.2%, 7.0% and 19.7%), respectively.
Jones-Rincon et al., 2019 [65]	US	Cross-sectional	3003/3361 (89.3%)	Elementary, middle/junior high or high	Patient Health Questionnaire. Job satisfaction was measured with 10 items.	Anxiety disorder: absenteeism, MDD, panic disorder, and somatization disorder and higher intent to quit, Hispanic, subject taught, job satisfaction and job control, years taught. teaching (*p* = 0.009).	65.8% major depression in the anxiety group and 11.2% major depression in the no anxiety group. Other depressive disorder among anxiety disorder group 8.4% and no-anxiety group 7.2%.
Borrelli et al., 2014 [133]	Italy	Cross-sectional	113/180 (63%)	Primary/middle	The Karasek Job Content Questionnaire, the Self-Rating Anxiety Scale (SAS) and the Center for Epidemiologic Studies Depression Scale (CES-D).	Depression and anxiety: Job demand and low social support.	About 50% scored above the threshold for depression and for anxiety on self-rating questionnaires.
Kinnunen et al., 1994 [51]	Finland	Survey	1012/1308/ (77%)	High/vocational/special/physical/secondary 45–59	Anxiety-contentment and depression-enthusiasm; six-item, six-point scales.	Job-related anxiety and depression: subject taught, age, job competence, and job aspiration, lack of PA. Physical education teachers, sex, poor work ability.	Not mentioned.
Martínez et al., 2020 [46]	Spain	Random Sampling	215/300 (71.7%)	Primary 30 to 65 years, *M* = 44.89	Zung Self-Rating Depression Scale (SDS), Coping with Stress Questionnaire.	Depressive symptomatology: quality of interpersonal relationships at school, dimensions of burnout.	Not mentioned.
Hadi et al., 2008 [94]	Malaysia	Cross-sectional	565/580 (97.4%)	Secondary M = 40.5	Depression, Anxiety and Stress Scale (DASS 21) and Job Content Questionnaire (JCQ).	Depression: decision latitude, psychological job demand and job insecurity.	The prevalence of depression was 49.1% (45.0, 53.2). Mild level of depression (21.0%).
Ali et al., 2021 [66]	Fiji.	Cross-sectional	SS = 375	Physical education 20 to 55 years	The Stress with COVID-19 Scale (SCS). The Coronavirus Anxiety Scale (CAS).	Anxiety: social support, and sexual satisfaction during the COVID-19 lockdown, marital status. Married physical education teachers experience more stress.	Married couples scored higher on stress. Anxiety and social support, single teachers scored high.
Capone et al., 2019 [70]	Italy	Cros-sectional	SS = 609	High school, middle school, elementary and primary school. 27 to 65, mean = 48.35	The Center for Epidemiologic Studies Depression Scale (Italian version. The Teacher Self-Efficacy Scale.	Depression: collective efficacy, all the dimensions of school climate were negatively related to depression, sex.	Women displayed higher depression and exhaustion than men.
Aydogan 2009 [84]	Turkey N = 83 Germany N = 78 Cyprus N = 74	Cross-sectional	SS = 235	High M = 38 ± 6.96, 37.9 ± 6.74, 45.8 ± 10.42	Depression, Anxiety stressTurkish version scale DASS-42.	Depression: burnout, country of origin, job satisfaction.	Not mentioned.
Kidger et al., 2016 [81]	Bristol, England	Cross-sectional	555/708/ (78.4%)	Secondary	Warwick Edinburgh Mental Wellbeing Scale-WEMWBS) Depressive symptoms (Patient Health Questionnaire-PHQ-9). Copenhagen Psychosocial Questionnaire and the Bristol Stress and Health at Work.	Depressive symptoms: sickness absence, student attendance, dissatisfaction with work and high presenteeism, gender, supporting a colleague. Teachers’ wellbeing.	19.4% moderate to severe depressive symptoms.
Bianchi et al., 2015 [99]	France	Survey	SS=627	Primary/secondary	Depression was assessed with the 9-item depression module.	Baseline depressive symptoms predicted cases of major depression.	T1 baseline MDD 14% T 2 MDD 7%.
Soria-Saucedo et al., 2018 [61]	Mexico	Cross-sectional	SS = 43,845	Female 25–74	Patient Health Questionnaire (PHQ9).	Severe depression: family and work stress, physical activity, alcohol consumption, and smoking, rural/urban residents.	7026 teachers (16%) severe depression.
Gluschkoff et al., 2016 [80]	Finland	Randomized selection	SS = 76	Primary/25–63	PHQ9.	Depressive symptoms: positive associations with effort–reward imbalance and job strain showed with depressive symptoms. Non-restorative sleep.	Not mentioned.
Ramberg et al., 2021 [91]	Sweden	Cross-sectional	Year 2014/16 3948/7147 (55.2%) Final SS = 2732	Teachers	Stockholm Teacher Survey.	Depressed mood: high SOC among colleagues and stress. High SOC was linked with lower levels of stress and depressed mood variation of 4.8% for perceived stress and 2.1% for depressed mood.	Not mentioned.
Pohl et al., 2022 [48]	Hungary	Cross-sectional	1817/2500 (72.7%)	High school/18–65	BDI.	Moderate and severe depression: internet addiction.	37.1%: no depression, 58.9% mild, 3.5% moderate and 0.6% severe depression.
Papastylianou et al., 2009 [3]	Greece	Cross-sectional	562/985 (57.1%)	Primary/30–45	The Centre for Epidemiologic Studies Depression scales.	Depressed affect: (positive) correlation emotional exhaustion (EE).	Depressed affect: 17.86%.
Ratanasiripong et al., 2021 [40]	Thailand	Cross-sectional	SS = 267	Primary/secondary	Depression, Anxiety and Stress Scale Thai Version (DASS).	Depression: family economics status, marital status, classroom size, relationship quality and resilience. Anxiety: family economics status, classroom size and resilience.	3.2% of teachers had severe to extremely severe depression, 11.2% had severe to extremely severe anxiety.
Szigeti et al., 2017 [50]	Hungary	Cross-sectional	SS = 211	Primary/secondary	Epidemiological Studies-Depression scale.	Depressive symptoms: teaching children with special needs, general burnout factor.	Not mentioned.
Baka 2015 [73]	Poland	Cross-sectional	316/400 (79%)	Primary/secondary 22–60	The Beck Hopelessness Scale.	Depression: work hours, job demands, general job burnout. High level of depression: interpersonal conflicts, organizational constraints and quantitative workload.	Not mentioned.
Othman et al., 2019 [42]	Malaysia	Cross-sectional	SS = 356	Secondary	Malay Depression Anxiety Stress Scales (DASS).	Depression, anxiety, and stress: socio-demographic and work-related characteristics such as female, spousal help, educational status, having 1–3 children.	Depression (43.0%), anxiety (68.0%), severe to extremely severe depression 9.9%, anxiety 23.3%. 84.6% depression among those educated up to secondary or diploma level. 45% and 47.6% teachers with longest teaching experience and highest income, respectively. Lack of spousal help (55%) depressed.
Skaalvik et al., 2020 [18]	Norway	Longitudinal	SS = 262	High school	Depressed mood was measured by means of a five-item scale.	Depressed mood: positively associated with emotional exhaustion.	Not mentioned.
Li et al., 2020 [55]	China	Cross-sectional	1741/1795 (97%)	Preschool 18 to 48	Epidemiologic Studies Depression Scale (CES-D) and the Perceived Stress Scale-14.	Depression: teacher weight. Depression (*p* < 0.001, OR = 3.08, 95% CI: 2.34–4.05) is significantly associated with burnout.	Depression was 39.9%.
Gosnell et al., 2021 [57]	Malaysia	Cross-sectional	124/400 (31%)	Primary/secondary	Depression Anxiety Stress Scales-21 self-care strategy questionnaire.	Depression/anxiety—self-care, being a refugee. Depression and anxiety: negative correlation with age. Younger teachers experienced higher rates of depression and anxiety than older teachers.	14.4% depression in the severe or extremely severe clinical ranges. 41.2% anxiety levels in the severe or extremely severe clinical ranges. 10.5% nonrefugees reported anxiety at this level.
Capone et al., 2020 [59]	Italy	Cross-sectional	SS = 285	High school 29–65	The Center for Epidemiologic Studies Depression Scale (CES-D; Italian version.	Depression: flourishing or languishing.	23.9% depression “flourishing” group, 38.7% low depression and burnout, 85.7% “languishing” had severe rating of depression.
Chan et al., 2002 [74]	China	Survey	SS = 83	Secondary 22–42	The shortened 20-item Teacher Stressor Scale (TSS). Chinese shortened version of the General Health Questionnaire (GHQ-20).	Anxiety: support, stress.	New teachers’ highest levels of symptoms in anxiety.
Zhang et al., 2014 [52]	China	Survey	SS = 590	Primary/secondary 34 ±8.11	Self-reported mental health was measured by the Symptom Checklist-90 (SCL-90).	Anxiety: burnout (EE and DP).	Not mentioned.
Nakada et al., 2016 [87]	Japan	Cross-sectional	1006 (66.7%)	School teachers 39.7 ± 11.6	The Japanese version of Zung’s Self-Rating Depression Scale (SDS), Job Stress Questionnaire.	Depressive symptoms: role ambiguity, role conflict, high quantitative workload, and social support from family or friends.	(20.1%) in depressive group. (79.9%) in non-depressive group.
Georgas et al., 1984 [79]	Greece	Cross-sectional	SS = 129	Elementary school teachers 28–46	Greek adaptation of the Schedule of Recent Experiences (SRE) Life Events Scale. The Manifest Anxiety Scale.	Anxiety: women only; psychosocial stress, sex differences, high correlations between psychosocial stress and anxiety, were found only for females.	Females reported more symptoms and had higher manifest anxiety than males.

Sample Size: SS; Major Depressive Disorder: MDD.
